# Supplementary material for: Peer-coaching interventions for stroke survivors - what works and how: A scoping review
Source: PLoS One. 2026 Apr 7;21(4):e0340169. doi: 10.1371/journal.pone.0340169 (PMC13056184; doi:10.1371/journal.pone.0340169)
Supplement: S1 Table — A table that lists database searches, keywords, and Boolean operators used in the literature search. (DOCX) [file pone.0340169.s001.docx]

**S1 Table. Search strategies.**

| Search number | Search term | Search result in October, 2025 |
| --- | --- | --- |
| Medline | | |
| 1 | Stroke/ or Stroke Rehabilitation/ or Stroke, Lacunar/ or Cerebrovascular Disorders/ | 200,873 |
| 2 | (stroke* or poststroke or post-stroke or apoplexy).ab,ti. | 370,437 |
| 3 | (stroke adj2 (patient? or survivor? or people or person? or individual?)).ab,ti. | 71,629 |
| 4 | (cerebrovascular and (accident* or disorder* or disease* or occlusion* or insufficienc*)).ab,ti. | 56,283 |
| 5 | 1 or 2 or 3 or 4 | 448,494 |
| 6 | Peer Group/ or Social Support/ or Peer Influence/ | 110,377 |
| 7 | peer?.ab,ti. | 161,886 |
| 8 | 6 or 7 | 248,389 |
| 9 | 5 and 8 | 2,740 |
| 10 | limit 9 to (english language and yr="1993 - 2025") | 2,598 |
| PubMed | | |
| 1 | ((("stroke"[MeSH:NoExp]) OR ("stroke rehabilitation"[MeSH:NoExp])) OR ("stroke, lacunar"[MeSH:NoExp])) OR ("cerebrovascular disorders"[MeSH:NoExp]) | 200,785 |
| 2 | stroke*[Title/Abstract] OR poststroke[Title/Abstract] OR post-stroke[Title/Abstract] OR apoplexy[Title/Abstract] | 380,632 |
| 3 | "stroke patient"[Title/Abstract:~2] OR "stroke patients"[Title/Abstract:~2] OR "stroke survivor"[Title/Abstract:~2] OR "stroke survivors"[Title/Abstract:~2] OR "stroke people"[Title/Abstract:~2] OR "stroke person"[Title/Abstract:~2] OR "stroke persons"[Title/Abstract:~2] OR "stroke individual"[Title/Abstract:~2] OR "stroke individuals"[Title/Abstract:~2] | 92,666 |
| 4 | cerebrovascular[Title/Abstract] AND (accident*[Title/Abstract] OR disorder*[Title/Abstract] OR disease*[Title/Abstract] OR occlusion*[Title/Abstract] OR insufficienc*[Title/Abstract]) | 62,749 |
| 5 | #1 OR #2 OR #3 OR #4 | 458,808 |
| 6 | (("peer group"[MeSH:NoExp]) OR ("social support"[MeSH:NoExp])) OR ("peer influence"[MeSH:NoExp]) | 110,332 |
| 7 | peer[Title/Abstract] OR peers[Title/Abstract] | 163,615 |
| 8 | #6 OR #7 | 249,933 |
| 9 | #5 AND #8 | 2,820 |
| 10 | #5 AND #8 AND (1993:2025[pdat])) | 2,753 |
| 11 | #5 AND #8 AND ((english[Filter]) AND (1993:2025[pdat])) | 2,678 |
| Embase | | |
| 1 | cerebrovascular accident/ or stroke rehabilitation/ or lacunar stroke/ or cerebrovascular disease/ | 561,794 |
| 2 | (stroke* or poststroke or post-stroke or apoplexy).ab,ti. | 603,339 |
| 3 | (stroke adj2 (patient? or survivor? or people or person? or individual?)).ab,ti. | 125,459 |
| 4 | (cerebrovascular and (accident* or disorder* or disease* or occlusion* or insufficienc*)).ab,ti | 86,744 |
| 5 | 1 or 2 or 3 or 4 | 816,460 |
| 6 | peer group/ or social support/ or peer pressure/ | 174,223 |
| 7 | peer?.ab,ti. | 206,398 |
| 8 | 6 or 7 | 344,075 |
| 10 | 5 and 8 | 5,867 |
| 11 | limit 9 to (english language and yr="1993 - 2025") | 5,698 |
| CINAHL | | |
| 1 | (MH "Stroke") OR (MH "Stroke, Lacunar") OR (MH "Cerebrovascular Disorders") | 88,792 |
| 2 | TI ( stroke* or poststroke or post-stroke or apoplexy ) OR AB ( stroke* or poststroke or post-stroke or apoplexy ) | 124,700 |
| 3 | TI ( stroke N2 (patient# or survivor# or people or person# or individual#) ) OR AB ( stroke N2 (patient# or survivor# or people or person# or individual#) ) | 38,370 |
| 4 | TI ( cerebrovascular AND (accident* or disorder* or disease* or occlusion* or insufficienc*) ) OR AB ( cerebrovascular AND (accident* or disorder* or disease* or occlusion* or insufficienc*) ) | 11,327 |
| 5 | S1 OR S2 OR S3 OR S4 | 152,1761 |
| 6 | (MH "Peer Group") OR (MH "Support, Social") OR (MH "Peer Pressure") | 31,493 |
| 7 | TI peer# OR AB peer# | 78,017 |
| 8 | S6 OR S7 | 94,691 |
| 9 | S5 AND S8 | 861 |
| 10 | S5 AND S8 (Limiters – Publication Date: 19930101-20251231; Narrow by Language: - English) | 833 |
| Cochrane Library | | |
| 1 | MeSH descriptor: [Stroke] this term only | 16,734 |
| 2 | MeSH descriptor: [Stroke Rehabilitation] this term only | 4,408 |
| 3 | MeSH descriptor: [Stroke, Lacunar] this term only | 71 |
| 4 | MeSH descriptor: [Cerebrovascular Disorders] this term only | 1,859 |
| 5 | (stroke* or poststroke or post-stroke or apoplexy):ti,ab,kw (Word variations have been searched) | 80,413 |
| 6 | (stroke NEAR/2 (patient? or survivor? or people or person? or individual?)):ti,ab,kw (Word variations have been searched) | 21,651 |
| 7 | (cerebrovascular AND (accident* or disorder* or disease* or occlusion* or insufficienc*)):ti,ab,kw (Word variations have been searched) | 26,606 |
| 8 | #1 OR #2 OR #3 OR #4 OR #5 OR #6 OR #7 | 87,678 |
| 9 | MeSH descriptor: [Peer Group] this term only | 2,115 |
| 10 | MeSH descriptor: [Social Support] this term only | 4,440 |
| 11 | MeSH descriptor: [Peer Influence] this term only | 76 |
| 12 | (peer?):ti,ab,kw (Word variations have been searched) | 19,054 |
| 13 | #9 OR #10 OR #11 OR #12 | 22,914 |
| 14 | #8 AND #13 with Cochrane Library publication date Between Jan 1993 and Dec 2025 | 657 |
| PsycINFO | | |
| 1 | (DE "Cerebrovascular Accidents") OR (DE "Cerebrovascular Disorders") | 31,532 |
| 2 | TI ( stroke* or poststroke or post-stroke or apoplexy ) OR AB ( stroke* or poststroke or post-stroke or apoplexy ) | 43,886 |
| 3 | TI ( stroke N2 (patient# or survivor# or people or person# or individual#) ) OR AB ( stroke N2 (patient# or survivor# or people or person# or individual#) ) | 15,610 |
| 4 | TI ( cerebrovascular AND (accident* or disorder* or disease* or occlusion* or insufficienc*) ) OR AB ( cerebrovascular AND (accident* or disorder* or disease* or occlusion* or insufficienc*) ) | 6,078 |
| 5 | S1 OR S2 OR S3 OR S4 | 52,157 |
| 6 | (DE "Social Support") OR (DE "Peer Pressure") | 74,606 |
| 7 | TI peer# OR AB peer# | 138,694 |
| 8 | S6 OR S7 | 205,707 |
| 9 | S5 AND S8 | 819 |
| 10 | S5 AND S8 (Limiters – Publication Date: 19930101-20251231; Narrow by Language: - english) | 756 |
