# Supplementary material for: Peer-coaching interventions for stroke survivors - what works and how: A scoping review
Source: PLoS One. 2026 Apr 7;21(4):e0340169. doi: 10.1371/journal.pone.0340169 (PMC13056184; doi:10.1371/journal.pone.0340169)
Supplement: S2 Table — A table that lists the quality assessment results of the included studies, with item-specific scores of each study. (DOCX) [file pone.0340169.s002.docx]

**S2 Table. Assessment results of included studies.**

|  | Kessler et al. 2014 | Kronish et al. 2014 | Sadler et al. 2017 | Masterson-Algar et al. 2020 | Hilari et al. 2021 | Moss et al. 2022 | Wan et al. 2024 | Rose et al. 2024 |
| --- | --- | --- | --- | --- | --- | --- | --- | --- |
| 1. Theoretical or conceptual underpinning to the research | 1 | 1 | 2 | 3 | 1 | 2 | 3 | 2 |
| 2. Statement of research aim/s | 3 | 3 | 2 | 3 | 3 | 2 | 3 | 2 |
| 3. Clear description of research setting and target population | 2 | 3 | 2 | 1 | 3 | 3 | 3 | 3 |
| 4. The study design is appropriate to address the stated research aim/s | 2 | 3 | 2 | 3 | 3 | 3 | 3 | 3 |
| 5. Appropriate sampling to address the research aim/s | 2 | 3 | 1 | 1 | 3 | 2 | 3 | 2 |
| 6. Rationale for choice of data collection tool/s | 1 | 3 | 2 | 1 | 3 | 3 | 3 | 1 |
| 7. The format and content of data collection tool is appropriate to address the stated research aim/s | 2 | 3 | 2 | 2 | 3 | 3 | 3 | 3 |
| 8. Description of data collection procedure | 2 | 3 | 2 | 2 | 3 | 3 | 2 | 1 |
| 9. Recruitment data provided | 2 | 3 | 2 | 2 | 3 | 1 | 3 | 1 |
| 10. Justification for analytic method selected | 2 | 3 | 1 | 2 | 2 | 0 | 3 | 0 |
| 11. The method of analysis was appropriate to answer the research aim/s | 3 | 3 | 2 | 2 | 3 | 3 | 3 | 2 |
| 12. Evidence that the research stakeholders have been considered in research design or conduct. | 2 | 1 | 2 | 3 | 2 | 3 | 0 | 1 |
| 13. Strengths and limitations critically discussed | 3 | 3 | 1 | 2 | 2 | 2 | 3 | 1 |
| Total score | 27 | 35 | 23 | 27 | 34 | 30 | 35 | 22 |
