# Supplementary material for: Peer-coaching interventions for stroke survivors - what works and how: A scoping review
Source: PLoS One. 2026 Apr 7;21(4):e0340169. doi: 10.1371/journal.pone.0340169 (PMC13056184; doi:10.1371/journal.pone.0340169)
Supplement: S3 Table — A table that lists detailed characteristics of the included interventions, including development process, component, theoretical basis, treatment window, peer recruitment and training, and mode of delivery. (DOCX) [file pone.0340169.s003.docx]

**S3 Table. Description of post-stroke peer-coaching interventions.**

| Author, year (country) | Development process | Component | Theoretical basis of components | Treatment Window | Peer inclusion/exclusion criteria | Peer training content | Mode of delivery |
| --- | --- | --- | --- | --- | --- | --- | --- |
| Kessler et al. 2014 (Canada) | Not reported | Provision of hope; provision of support and information | Not reported | Starting from hospitalisation with post-discharge follow-ups | Not reported | Coaching procedures, coach role, discussion topics, hospital orientation, session shadowing | **Format**: individual-based  **Setting**: hospital and remote  **Dosage**: 6 sessions in 12 months  **Session length**: 5-60 minutes |
| Kronish et al. 2014 (US) | Adapted from an existing program with PAR | Self-management modelling; problem-solving; action planning; progress feedback; social persuasion | Not reported | Within 5 years post-stroke | Not reported | Training based on CDSMP philosophy and methods | **Format**: group-based  **Setting**: community  **Dosage**: 6 sessions in 6 weeks  **Session length**: 1.5 hours |
| Sadler et al. 2017 (UK) | Co-designed with stakeholders | Meaning making; social support; information support; strategy and long-term management training | Social learning theory | 6-24 months post-stroke | Inclusion: a good level of adjustment and recovery following stroke | Not reported | **Format**: group-based  **Setting**: community  **Dosage**: 12 sessions in 6 weeks  **Session length**: 50 minutes |
| Masterson-Algar et al. 2020 (UK) | Co-designed with stroke survivors and peer coaches | Connecting and sharing, goal setting, action planning, reflecting | Theory of planned behaviour; self-efficacy theory; common sense model of illness self-regulation | Within 6 months post-stroke; post-discharge | Not reported | Coach role, coaching content, theoretical principles, effective communication | **Format**: individual-based  **Setting**: public settings  **Dosage**: 6 sessions  **Session length**: up to 1 hour |
| Hilari et al. 2021; Moss et al. 2022 (UK) | Developed based on an existing scheme with active PCPI | Goal setting, conversation, problem solving, and joint activities | Not reported | Early-stage post-stroke after hospitalization and intensive rehabilitation | **Inclusion**: mild-moderate aphasia, ≥ 1-year post-stroke  **Exclusion**: diagnosed with conditions effecting cognition or mental health; severe uncorrected visual or hearing problems; severe or potentially terminal co-morbidities; discharged outside of the borough of the recruiting hospital | Coach role, coaching content, communication techniques, health and safety, and management of adverse event | **Format**: individual-based  **Setting**: participants' home or community  **Dosage**: 6 sessions in 3-4 months (with 2 optional sessions)  **Session length**: 1 hour |
| Wan et al. 2024 (China) | Not reported | Didactic education, emotional management, self-management strategy, and social participation strategies | Person-Environmental-Occupation-Performance model | Not reported | Not reported | Knowledge acquisition, skill development, experiential learning | **Format:** group-based **Setting:** community health centres, family doctors' studios, or rehabilitation units **Dosage:** 6 sessions in 6 weeks **Session length:** 2 hours |
| Rose et al. 2024 (Australia) | Developed based on an existing programme | Multi-modal communication, information about aphasia and living well, emotional support, activities for fostering positive identity, and optional components | Not reported | After 6 months post-stroke | Inclusion: member of the community Not requirement on relevant qualifications or experience | Description of community aphasia groups; rationale for peer-led groups; facilitation skills; group programme activity options | **Format:** group-based **Setting:** not reported **Dosage:** 12 sessions in 12 weeks **Session length:** 2 hours |
