# Supplementary material for: Peer-coaching interventions for stroke survivors - what works and how: A scoping review
Source: PLoS One. 2026 Apr 7;21(4):e0340169. doi: 10.1371/journal.pone.0340169 (PMC13056184; doi:10.1371/journal.pone.0340169)
Supplement: S4 Table — A table that lists the detailed outcomes of quantitative investigations and qualitative inquiries conducted in the included studies. (DOCX) [file pone.0340169.s004.docx]

**S4 Table. Description of intervention outcomes.**

| **Reference** | **Intervention outcomes** |
| --- | --- |
| **Quantitative Investigation** | |
| Kronish et al. 2014 | No difference in the proportion of participants with controlled cholesterol, and those taking antithrombotic medication; a higher proportion of participants with controlled blood pressure in the intervention group at 6 months post-intervention. |
| Sadler et al. 2017 | Post-intervention improvement in resilience, quality of life (QoL) without a significance level provided; no difference in activity level and depression. |
| Hilari et al. 2021 | No significant difference in general well-being, psychological well-being, friendship and communication; a higher level of community integration in the control group. |
| Wan et al. 2024 | Greater improvement in social participation, participation self-efficacy, perceived social support, and QoL in the intervention group; greater decrease in psychological distress and disease stigma in the intervention group. |
| Rose et al. 2024 | No confident finding due to a limited sample size and external confounding factors; detectable improvement in overall QoL and communication-related QoL in 4 of the 7 participants. |
| **Qualitative Inquiry** | |
| Kessler et al. 2014 | The intervention perceived as beneficial and offering emotional, affirmational and informational support; no harm indicated by stroke survivors; concerns expressed by caregivers and professionals. |
| Sadler et al. 2017 | The intervention perceived as beneficial and acceptable. |
| Masterson-Algar et al. 2020 | Limited qualitative data collected on intervention recipients’ perceptions. |
| Moss et al. 2022 | The intervention perceived as beneficial on emotional well-being; participants satisfied with intervention logistics . |
| Rose et al. 2024 | The intervention perceived as positive and beneficial and considered as a safe place for connection and participation. |
