# Supplementary material for: Peer-coaching interventions for stroke survivors - what works and how: A scoping review
Source: PLoS One. 2026 Apr 7;21(4):e0340169. doi: 10.1371/journal.pone.0340169 (PMC13056184; doi:10.1371/journal.pone.0340169)
Supplement: Attachment — Submitted filename: Response to Reviewers.docx [file pone.0340169.s007.docx]

Doctor Yilin Jiang

Academic Editor

*PLOS One*

10^th^ February 2026

Dear Doctor Jiang,

Thank you for the opportunity to revise our manuscript entitled, “Peer-coaching Interventions for Stroke Survivors - What Works and How: A Scoping Review.” We would also like to express our gratitude to the reviewers for their insightful comments and constructive suggestions, which have helped us improve the clarity and quality of our work significantly.

We have addressed all the points raised by the editorial team and the reviewers. A point-by-point response is provided below, and all changes have been highlighted in the “Revised Manuscript with Track Changes” file.

**1. Responses to Comments from Academic Editor**

**Comment 1:** Please ensure that your manuscript meets PLOS ONE's style requirements, including those for file naming.

**Comment 2:** Please remove your figures from within your manuscript file, leaving only the individual TIFF/EPS image files, uploaded separately. These will be automatically included in the reviewers’ PDF.

**Author response:** Thank you for this guidance. We have examined the style of the materials and made necessary changes: correcting the format of the manuscript, removing the figure from the manuscript, correcting the file name and format of the figure file, and correcting the file names of supporting information files.

**2. Responses to Comments from Reviewer #1**

**Comment 1:** The authors adopted the “Working definition” from Ljungberg et al. (2011) for their literature search. However, they proposed a “standardised definition” as the final outcome. In the Methods section, it is stated that interventions not matching this working definition were excluded, raising concerns that this procedure may have resulted in the omission of studies that should have contributed to conceptualizing “post-stroke peer-coaching.” In the Discussion section, I recommend addressing, either as a limitation or as an addition to the Methodology, how (or if) filtering with the working definition influenced the final scope setting and generation of the definition.

**Author response:** We thank the reviewer for this critical observation. The working definition was applied to ensure the basic conceptual alignment across the included studies, which focused on interventions that involved stroke survivors as the primary support provider and introduced directional support to the service user. Specifically, it functioned as a systematic filter to differentiate the targeted interventions from other interventions that involved limited peer-based support or introduced support groups with the focus on mutual support between stroke survivors. According to our screening process and record, we believe that the articles that were excluded based on the working definition would not contribute to the development of intervention definition and the current work. However, we acknowledge that this measure of inclusion/exclusion still carries a risk of study omission. Following your guidance, we have indicated the application of the working definition as a limitation of the current work (Page 26). In addition, to address the concern of readers, we have clarified the number of articles that were excluded based on the working definition, as well as the reasons that these articles were excluded (Page 9).

**Comment 2:** Table 1 presents the characteristics of the included studies, and I understand that detailed information on each element designated as the scope—“definitions of interventions,” “characteristics,” and “outcomes”—is provided in Supplemental Materials 3 and 4. However, in the Results section (Intervention Characteristics), I believe that more frequent referencing of Table 1 and the Supplemental Materials would facilitate easier cross-referencing of the main text with the tables for readers.

**Author response**: We agree that more frequent reference to the tables will facilitate the reading process. Therefore, we have added several phrases that refer to Table 1 and tables from the supporting information across the result section (Line 188, 190, 197, 220, 222, 254, 274, 298, 304, and 322) .

**3. Responses to Comments from Reviewer #2**

**Author response:** We thank the reviewer for the positive assessment of our methodology and for recognising the value of the proposed definition.

**4. Responses to Comments from Reviewer #3**

**Comment 1 :** Please clarify how the working definition (based on Ljungberg et al.) may have influenced study inclusion and whether this could have excluded relevant peer-based interventions using alternative conceptualisations.

**Author response:** We thank the reviewer for the suggestions on clarifying the influence of the working definition, which was also raised by Reviewer#1. Following your guidance, we have clarified the number of articles that were excluded based on the working definition and the reasons that these articles were excluded (Page 9). We believe that the articles that were excluded based on the working definition would not contribute to the development of intervention definition and the current work. However, considering the potential risk of study omission, we have indicated the application of the working definition as a limitation of the current work (Page 26).

**Comment 2:** The decision to include studies from 1993 onward should be briefly justified. **Author response:** We have added this justification on Page 7, Line 128.

**Comment 3:** Although quality appraisal is not mandatory for scoping reviews, the implications of including lower-quality studies should be discussed more explicitly when interpreting findings.

**Author response:** We agree that the quality of included studies needs to be further discussed. We have added a more comprehensive summary of study quality in the discussion section (Page 20) and discussed the influence of study quality on the findings (Page 25)

**Comment 4:** The discussion should further address how cultural, healthcare system, and community differences may influence peer-coaching implementation and outcomes.

**Author response:** We have added a section that explains how the established definition can be employed to address different needs for support across diverse regions (Page 27), as the definition incorporates participatory action research, which can provide space for intervention tailoring.

**4. Responses to Comments from Reviewer #4**

**Comment 1:** In the introduction, I suggest minor adjustments to improve the initial understanding of the intervention addressed in the study. At first glance, before a full reading of the manuscript, the approach adopted in the review is not clearly described, which may limit the reader’s understanding of the study’s scope. The objectives are clearly presented; however, a clearer definition of the approach and scope of the review could enhance the clarity and accessibility of this section.

**Author response:** We thank the reviewer for the detailed and critical observation. We have added a brief definition to introduce scoping review as the study design in the introduction section (Page 5).

**Comment 2:** As a suggestion for improvement, I recommend a clearer definition of the inclusion criteria (for example, specifying the age of participants included in the analyzed studies), as well as the exclusion criteria.

**Author response:** We have added participant age as one of the inclusion criteria and structured the inclusion/exclusion criteria to improve the readability (Page 7).

**Comment 3:** I suggest that the information presented on page 17, lines 320 to 324, regarding the types of study designs of the included articles, also be incorporated into the results section, thereby strengthening the presentation of the findings.

**Author response:** We have added the information presented between line 320 and 324 into the results section (Page 10, Line 195 to 197).

**Comment 4:** Throughout the text, there are minor punctuation issues that could be addressed to further improve clarity

**Author response:** We have carefully examined the manuscript and corrected punctuation issues throughout the text.

We hope that these revisions adequately address the concerns raised and that the manuscript is now qualified for publication in *PLOS One*. We look forward to hearing from you.

Yours sincerely,

Andy HauYan HO, Ph.D., Ed.D.
Professor of Psychology, School of Social Sciences

Nanyang Technological University, Singapore
14 Nanyang Drive, HSS-04-03, Singapore 637332

Office No.: +65 63168943 Fax No.: +65 67955797 Email: [andyhyho@ntu.edu.sg](mailto:andyhyho@ntu.edu.sg)
